# Rethinking of ureteral stent removal using an extraction string; what patients feel and what is patients' preference? : a randomized controlled study

**DOI:** 10.1186/s12894-015-0114-6

**Published:** 2015-12-09

**Authors:** Dae Ji Kim, Jeong Hwan Son, Seok Heun Jang, Jae Won Lee, Dae Sung Cho, Chae Hong Lim

**Affiliations:** Department of Urology, Bundang Jesaeng Hospital, 180 Seohyeon-rho Bundang-gu, Seongnam, 463-774 Republic of Korea

**Keywords:** Urolithiasis, Ureteral stent, Stent removal, Pain, Preference

## Abstract

**Background:**

Ureteral stent removal using an extraction string is advantageous because it can obviate an invasive cystoscopy, but there is a paucity of data on how patients feel about it, and how bothersome or beneficial it is.

We performed this study to evaluate patients’ preference for stent removal using an extraction string and which parameters could affect it.

**Methods:**

In total, 114 consecutive patients undergoing ureteral stent insertion after ureteroscopic stone removal (URS) for unilateral recurrent ureter stones were enrolled. Patients were randomized to a string group or a no string group.

Stent removal was performed on the first visit within 7 days postoperatively. All patients were asked to complete the ureteral stent symptom questionnaire, to rate the degree of pain during stent removal using a visual analog scale (VAS) and to answer to questions regarding their preference.

**Results:**

No significant differences were found in domain total scores including urinary symptoms (p = 0.17), pain (p = 0.62), general health (p = 0.37), work performance (p = 0.41). However, regarding separate questions for ‘dysuria’ and ‘difficulties with heavy physical activity’, there were significant intergroup differences (p = 0.03 and p = 0.04, respectively). Particular, a significantly higher proportion of patients in the string group checked ‘stoppage of sexual intercourse due to stent-related problems’ than in the no string group (p = 0.03).

VAS score on stent removal was significantly higher in the no string group than the string group (p = 0.005).

Among the patients who remember the experience of an indwelling ureteral stent in the past, 85 % (17/20) of the no string group answered ‘No’ to the question of ‘difference between the methods used in this time and in the past’. On the contrary, 84.2 % (16/19) answered ‘Yes’ to the same question in the string group. And, all 16 patients of the string group who noted differences between the methods preferred ureteral stent removal using an extraction string to the past method.

**Conclusions:**

Despite of minor increased morbidity related to the extraction string, patients preferred ureteral stent removal using the extraction string after URS. The patients with the extraction string felt less pain on stent removal than flexible cystoscopic stent removal.

**Trial registration:**

KCT0001700. The trial was registered in the Clinical Research

Information Service (CRiS), Republic of Korea; registration date: 18/11/2015.

## Background

Removing a ureteral stent using an extraction string was first described by Siegel et al. in 1986 as a simple method to avoid general anesthesia and unnecessary urethral instrumentation for pediatric patients [1]. Subsequently, several descriptions of a method to remove ureteral stents using an extraction string have been published [2–6].

The method is advantageous because it can obviate an invasive cystoscopic procedure, but there is wide variability in its clinical application and a paucity of data on how many urological surgeons use extraction strings, how patients feel about them, and how bothersome or beneficial they are.

The ureteral stent is an integral armamentarium in the urologic surgical field. However, urologists must understand the morbidities related to removing a ureteral stent and the patients’ perception of stent removal as well as ureteral stent *in situ*-related morbidities.

The primary objective of this study was to evaluate patients’ preference for removing a ureteral stents using an extraction string. A secondary objective was to evaluate parameters that could affect patients’ preference using a visual analog scale (VAS) for pain during ureteral stent removal and the Ureteral Stent Symptom Questionnaire (USSQ) with respect to their ureteral stent *in situ*.

## Methods

In total, 114 consecutive patients undergoing insertion of ureteral stent after ureteroscopic stone removal (URS) between July 2012 and November 2014 were enrolled. This study was approved by Bundang Jesaeng General Hospital Institutional Review Board. Consent was obtained from all patients after providing with verbal and written information about the study.

Inclusion criteria were patients who had a double J ureteral stent inserted after URS for unilateral recurrent ureteral stones. Exclusion criteria were coexisting non-calculous disease (e.g., malignant obstruction, renal insufficiency, or congenital anomaly of urinary tract), solitary kidney, ureteral stricture, pregnancy, or complicated URS requiring long-term stent placement (>7 days). Patients who were taking an alpha-blocker or anti-cholinergic agent to treat lower urinary tract symptoms or who were taking analgesics for chronic pain were also excluded to rule out any influence of the drugs on the symptom questionnaire results.

**Fig. 1:**
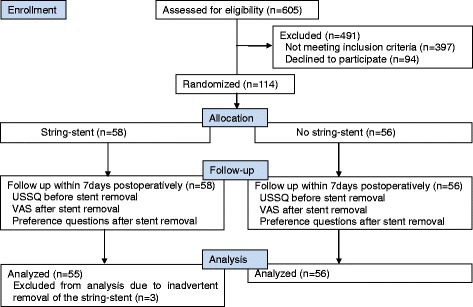
CONSORT flowchart for this study

After completing the URS, patients who met the inclusion criteria were randomized into the string-stent group or the no string-stent group (Fig. 1). Group allocation was performed using the random-number generator in Excel 2010 on an operating room computer before stent insertion.

All stents (6-F Percuflex plus; Boston Scientific, Natick, MA, USA) were inserted via a retrograde approach under cystoscopic guidance. Stent lengths were determined based on patient height. The stent string was manipulated to leave a new air knot 1 cm from the stent end, as described by Bockholt et al. [3]. The distal end of the string (4–5 cm long) was left protruding from the urethral meatus without securing it to the skin.

All patients were discharged the day following the operation with prescriptions for prophylactic antibiotics and non-steroidal anti-inflammatory drugs for several days until the first visit. Alpha-blockers and anti-cholinergics were not administered. The string-stent group patients were reminded to be cautious regarding the string to prevent inadvertent extraction.

Stents were removed in the outpatient department on the first visit within 7 days postoperatively. All patients were asked to complete the validated Korean version of the USSQ [7] immediately before the stent was removed. Urology residents removed the string-stents by pulling the string out without use of lidocaine jelly or an analgesic before the procedure. The no string-stents were removed by urology residents through flexible cystoscopic procedures in which 2 % lidocaine jelly was applied in the urethra without an analgesic. The patient was asked to rate the degree of pain during stent removal on a 10-cm VAS (Fig. 1).

We asked the following four questions regarding their preference:

“Have you ever had an operation to treat urolithiasis?”

“Do you remember your experience with a ureteral indwelling stent?”

“Was there a difference between the method used this time and the one used in the past regarding ureteral stent maintenance and removal?”

**Fig. 2:**
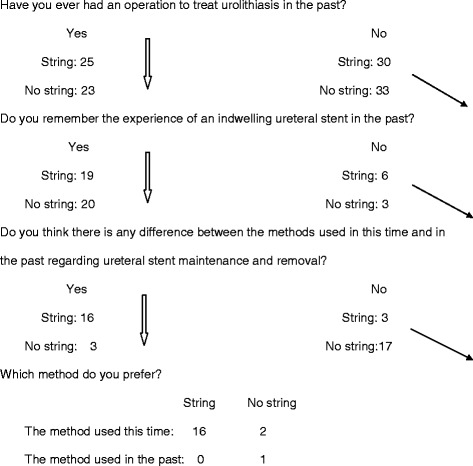
Flow diagram of preference questions (String: *n* = 55, No string: *n* = 56)

“Which method do you prefer?” (Figs. 1 and 2)

Sample size was calculated based on the results of previous ureteral stent studies. A sample size of 50 patients in each group was sufficient for 80 % power to detect a 20 % difference in each USSQ domain score.

Statistical analyses were performed using SPSS ver. 19.0 software (SPSS Inc., Chicago, IL, USA). Numerical data were compared using Student’s *t*-test. Categorical data were analyzed using the χ^2^ test. Statistical significance was set at P < 0.05.

## Results

In total, 114 patients were randomized into 58 patients in the string-stent and 56 in the no string-stent groups. No differences were observed for age, stone size, laterality, stone location, or *in situ* ureteral stent duration between the groups (Table 1).

**Table 1:** Patient characteristics

Characteristic	String	No string	P-value
Patients(n)	58	56	
Male:Female	42:16	36:20	0.34
Age(years)	50.97±12.20	50.54±14.28	0.86
Stone size(mm)	6.83±2.04	7.64±1.68	0.32
Laterality (n) Right:Left	28:30	27:29	0.85
Stone location(n) upper: mid: lower	4:12:42	7:12:37	0.53
Stent duration(days)	5.97	6.28	0.12

The USSQ was completed by 89 of 114 patients; 43 in the string group and 46 in the no string group. The VAS and the preference questions were completed by all patients except three patients who had suffered inadvertent removal of the string-stent before the first outpatient clinic visit.

Overall ureteral stent *in situ*-related symptoms are shown in Table 2. No significant differences were found in domain total scores, including urinary symptoms, pain, general health, or work performance. However, significant differences were observed between the groups for separate questions on “dysuria” and “difficulties with heavy physical activity” (2.96 vs. 2.36, p = 0.03, and 2.77 vs. 2.18, p = 0.04, respectively). In particular, all patients who completed the USSQ, except one in the string group, answered “no active sexual life”. Among them, 22 patients checked “stopped sexual intercourse after insertion of stent ”, which was “due to a stent-related problem” in 17 patients and was significantly higher than that in the no string group (77 % vs. 44.4 %, p = 0.03).

**Table 2:** Overall ureteral stent in situ-related symptoms

Domains	Items	Scores		Standard deviation		P
		String	No string	String	No string	
Urinary symptoms	Domain total	30.6	28.2	8.54	7.25	0.17*
	Daytime frequency	3.02	3.37	1.14	1.25	0.17*
	Nocturia	2.58	2.40	1.07	1.30	0.48*
	Urgency	2.21	1.97	1.01	1.11	0.31*
	Urge incontinence	1.42	1.33	0.63	0.82	0.59*
	Non urge incontinence	1.37	1.16	0.72	0.63	0.14*
	Residual urine sensation	3.02	2.60	1.18	1.29	0.11*
	Dysuria	2.95	2.36	1.53	1.05	0.03*
	Hematuria frequency	3.12	2.80	1.61	1.27	0.31*
	Hematuria amount	2.32	2.29	1.06	0.87	0.86*
	Interference in life	2.81	2.44	1.31	1.12	0.16*
	Quality of life impact	5.76	5.51	1.50	1.31	0.40*
Pain	Domain total	21.02	20.40	6.39	5.38	0.62*
	Presence or absence of pain	1.23	1.16	0.43	0.37	0.39*
	VAS	6.27	5.71	2.84	2.90	0.36*
	Pain associated with amount of physical activities	3.20	3.02	1.25	1.32	0.50*
	Sleep disturbance	2.20	2.13	1.10	1.04	0.74*
	Pain at voiding	3.33	3.09	1.24	1.04	0.34*
	Flank pain at voiding	1.37	1.47	0.49	0.50	0.37*
	Frequency of painkiller	2.16	2	1.13	0.93	0.46*
	Overall bother	3.05	2.84	1.31	1.04	0.42*
General health	Domain total	15.12	14.04	5.97	5.09	0.37*
	Difficulties with light physical activity	2.23	1.91	1.36	0.99	0.20*
	Difficulties with heavy physical activity	2.77	2.18	1.46	1.21	0.04*
	Feeling tired	2.33	2.4	1.21	1.18	0.77*
	Feeling calm and peaceful	3.40	3.53	1.43	1.42	0.65*
	Social life enjoyment	2.88	2.62	1.33	1.34	0.36*
	Need extra help	1.51	1.4	0.91	0.86	0.56*
Work performance	Domain total	14.41	15.6	6.32	7.04	0.41*
	Type of employment	2.30	3.31	2.22	2.65	0.06*
	Number of days bed rest all day long	1.79	1.47	2.73	2.30	0.55*
	Number of days reduced more than half daily activity	1.80	2.24	1.80	2.64	0.30*
	Position or role in workplace	1.81	1.75	0.93	0.84	0.74*
	Frequency of rest	1.98	1.08	2.44	1.22	0.06*
	Stent related changes on work	2.28	1.98	1.22	1.05	0.22*
	Changes in work duration	2.51	2.44	1.45	1.27	0.82*
Sexual matters						
	No active sexual life: n (%)	42(97.6)	38(82.6)	-	-	0.02**
	Stop of sexual Intercourse after stent: n (%)	22(52)	18(47.3)	-	-	0.82**
	Stop of sexual intercourse due to stent-related problem: n (%)	17(77)	8(44.4)	-	-	0.03**
Additional problems	Need for additional antibiotics (%)	0	0	-	-	-
	Admission or additional therapeutic procedure(%)	0	0	-	-	-

* Student’s *t*-test, ** χ^2^ test.

Male patients in the string and no string groups showed significant higher urinary symptom scores (33.14 vs. 25.87, p = 0.006, and 29.69 vs. 26.26, p = 0.012). No differences were found between the two sex subgroups in the other domains of pain, general health, or work performance (Table 3).

**Table 3:** Comparison of ureteral stent in situ-related symptoms between male and female subgroups

Domains		String	No string	P-value^a^
Urinary symptoms	Overall	30.60 ± 8.54	28.20 ± 7.25	0.17
	Male	33.14 ± 7.59	29.69 ± 6.79	0.08
	Female	25.87 ± 8.43	26.26 ± 7.56	0.89
	P-value^b^	0.006	0.012	
Pain	Overall	21.02 ± 6.39	20.40 ± 5.38	0.62
	Male	23.14 ± 6.08	21.58 ± 6.43	0.36
	Female	22.27 ± 7.79	21.16 ± 5.51	0.63
	P-value^b^	0.69	0.82	
General health	Overall	15.12 ± 5.97	14.04 ± 5.09	0.37
	Male	14.93 ± 4.98	13.50 ± 5.42	0.32
	Female	15.47 ± 7.68	14.79 ± 4.64	0.75
	P-value^b^	0.78	0.41	
Work performance	Overall	14.41 ± 6.32	15.60 ± 7.04	0.41
	Male	13.68 ± 6.24	14.50 ± 6.93	0.65
	Female	15.80 ± 6.47	17.11 ± 7.12	0.58
	P-value^b^	0.30	0.22	

^a^between string group and no string group, ^b^between male and female

The VAS scores are presented in Table 4. Overall, the mean pain score was 2.94 in those with string-stents and 4.23 in those with no string-stents who underwent the flexible cystoscopic removal procedure (p = 0.005).

**Table 4:** VAS pain scores on ureteral stent removal

VAS score	String	No string	P-value*
Overall	2.94±1.35	4.23±2.45	0.005
Male	3.19±1.09	4.58±2.23	0.006
Female	2.46±1.71	3.54±2.82	0.25
P-value**	0.22	0.11	

*between string group and no string group, **between male and female

As shown in Fig. 2, among patients who remembered their experience with an indwelling ureteral stent in the past, 85 % (17/20) in the no string group answered “No” to the question of a “difference between the method used this time and the one used in the past”. In contrast, 84.2 % (16/19) answered “Yes” to the same question in the string group. All 16 patients in the string group who noted a difference between the methods preferred removal of their ureteral stent using an extraction string compared to the method used previously.

No significant complications were noted, except three cases of inadvertent removal of the string-stent before the first outpatient clinic visit, none of which required replacement. No patient suffered from a febrile urinary tract infection requiring additional antibiotic treatment or a therapeutic procedure (Table 2).

## Discussion

The ureteral stent has long been an integral part of urology, as it reduces postoperative complications, such as stricture, urine leakage, and renal colic due to edema of the ureter [8]. However, various *in situ*-related ureteral stent problems, such as flank pain, hematuria, and lower urinary tract symptoms, can develop [9, 10]. Many studies have assessed how *in situ*-related stent discomfort can be alleviated, how long ureteral stents should be in-place, and the possibility of eliminating use of a ureteral stent after a ureteroscopic procedure. These studies consistently show that medications, such as alpha-blocker and anti-cholinergic agents, only alleviate some *in situ*-related ureteral stent symptoms. [11–16] Moreover, ureteral stent indwelling durations have been shortened [17, 18] and an indwelling ureteral stent is not required under certain conditions [19–21].

In addition to *in situ*-related ureteral stent problems, urologists must pay more attention to ureteral stent removal procedures, which are troublesome for patients. However, few studies have investigated alleviating *in situ*-related ureteral stent discomfort. Several reports have described endoscopic and non-endoscopic methods intended to reduce pain during cystoscopic stent removal or provide a substitute for the cystoscopic procedure. The ureteral stent extraction string is an alternative that can obviate the need for a cystoscopic procedure [1–6, 22, 23].

Most ureteral stents from various manufacturers have a string connected to the stent, which is used for removing or intraoperative repositioning of a stent. Some surgeons believe the string is useful to avoid an invasive cystoscopic procedure to remove a stent and reduce cost. However, others hesitate to use a string-stent due to possible inadvertent removal or increased stent-related discomfort or complications. Our result that 84.2 % of the string group found a difference between methods used this time and previously and 85 % of the no string group did not find any difference suggests that the extraction string had not been used popularly in our study cohort.

In a recent study on ureteral stent extraction strings, Barnes et al. [4] reported that the index scores for the USSQ domains of urinary symptoms, pain, general health, and work performance were not different between the groups and that use of a stent extraction string after URS for stone disease did not increase stent-related urinary symptoms, complications, or morbidity during removal.

In addition to such parameters, our study focused on evaluating patients’ preference for ureteral stent removal using an extraction string. As results, we noted that most patients preferred removal of the ureteral stent using an extraction string, although there were concerns about the impact on their sex life and minor increases in stent-related urinary symptoms, such as dysuria and difficulties with heavy physical activity. The general domain scores on the USSQ were not different between the two groups, except for the questions on “dysuria” and “difficulties with heavy physical activity”, suggesting that the stent extraction string only slightly increased *in situ*-related stent urinary symptoms or complications.

Male patients showed significantly higher urinary symptom scores in both groups, suggesting that males tend to suffer more from urinary symptoms related to a ureteral stent *in situ*. This may also result from anatomic differences between males and females, for example with respect to the prostate gland and a longer urethra.

Overall VAS scores were different between the string and no string groups but were more marked in male than female patients. Barnes et al. [4] reported no difference in VAS pain scores of patients during stent removal (2.5 in those with a stent string and 3.1 in those with no string using cystoscopy, p = 0.45). In a small study that used a VAS to evaluate pain during stent removal using rigid cystoscopy, Kuehhas et al. [24] found no difference in pain between patients undergoing cystoscopy and extraction using a stent string. These results differ from our VAS scores and may have been influenced by the conditions of our cystoscopic stent removal procedure, such as use of lidocaine jelly but not an analgesic. In addition, different clinical experiences may have caused these differences, as Kuehhas et al. [24] reported that clinical experience is correlated with pain scores of cystoscopic procedures. In fact, mean VAS scores for cystoscopic stent removal were 1.87–8 in several studies. This broad range is thought to be due to heterogeneous settings, such as type of cystoscope or whether any adjunctive medications or local anesthesia was used [4, 24–26].

A few pain-related studies have reported cystoscopic removal of stents, but the results were obtained under heterogeneous settings. Large, controlled studies on pain related to cystoscopic stent removal are warranted.

Loh-Doyle et al. [26] investigated patient experiences of, and preference for, removing ureteral stents through an anonymous website-based survey that included a large sample size and reported the following results by method : “willingness to undergo the same procedure again”; cystoscopy in clinic in 51 %, doctor’s office pulled string in 55 %, cystoscopy in the operating room in 67 %, and pulling the string myself in 60 %. We acknowledge the small sample size of our study and that it was aimed at patients with recurrent ureteral stones. However, our study had a relative strength, as it presented the preferences of a patient cohort that experienced several procedures. The second limitation was that patients only had stents in-place for only 7 days after URS. So, whether this method is useful in cases that require a stent for a longer period, such as URS for complicated pyelonephritis, traumatic URS, ureteroscopic surgery for a ureteral stricture, or periodic stent changes for a malignant ureteral stricture, is unknown. Additional studies are necessary to determine perceptions and related morbidities in patients who have an indwelling ureteral stent and extraction string for a longer period.

## Conclusions

Despite a minor increase in morbidity related to the extraction string, patients preferred removal of their ureteral stent using an extraction string after URS. The patients in the extraction string group felt less pain when the stent was removed than when it was removed with a flexible cystoscope.

However, appropriate counseling for the minor increase in morbidities is needed before applying the method clinically. In particular, concerns related to sexual activity should be considered for patients with an active sexual life.

## Abbreviations

VAS: Visual analog scale
USSQ: Ureteral Stent Symptom Questionnaire
URS: Ureteroscopic stone removal
